# Pituitary Adenylate Cyclase Activating Polypeptide (PACAP) Signalling Exerts Chondrogenesis Promoting and Protecting Effects: Implication of Calcineurin as a Downstream Target

**DOI:** 10.1371/journal.pone.0091541

**Published:** 2014-03-18

**Authors:** Tamás Juhász, Csaba Matta, Éva Katona, Csilla Somogyi, Roland Takács, Pál Gergely, László Csernoch, Gyorgy Panyi, Gábor Tóth, Dóra Reglődi, Andrea Tamás, Róza Zákány

**Affiliations:** 1 Department of Anatomy, Histology and Embryology, University of Debrecen, Faculty of Medicine, Debrecen, Hungary; 2 Cell Biology and Signalling Research Group of the Hungarian Academy of Sciences, Department of Medical Chemistry, Research Centre for Molecular Medicine, Faculty of Medicine, University of Debrecen, Debrecen, Hungary; 3 Department of Physiology, University of Debrecen, Faculty of Medicine, Debrecen, Hungary; 4 Department of Biophysics and Cell Biology, University of Debrecen, Faculty of Medicine, Debrecen, Hungary; 5 Department of Medical Chemistry, University of Szeged, Faculty of Medicine, Szeged, Hungary; 6 Department of Anatomy PTE-MTA “Lendület” PACAP Research Team, University of Pécs, Medical School, Pécs, Hungary; Universidad de Castilla-La Mancha, Spain

## Abstract

Pituitary adenylate cyclase activating polypeptide (PACAP) is an important neurotrophic factor influencing differentiation of neuronal elements and exerting protecting role during traumatic injuries or inflammatory processes of the central nervous system. Although increasing evidence is available on its presence and protecting function in various peripheral tissues, little is known about the role of PACAP in formation of skeletal components. To this end, we aimed to map elements of PACAP signalling in developing cartilage under physiological conditions and during oxidative stress. mRNAs of PACAP and its receptors (PAC1,VPAC1, VPAC2) were detectable during differentiation of chicken limb bud-derived chondrogenic cells in micromass cell cultures. Expression of PAC1 protein showed a peak on days of final commitment of chondrogenic cells. Administration of either the PAC1 receptor agonist PACAP 1-38, or PACAP 6-38 that is generally used as a PAC1 antagonist, augmented cartilage formation, stimulated cell proliferation and enhanced PAC1 and Sox9 protein expression. Both variants of PACAP elevated the protein expression and activity of the Ca-calmodulin dependent Ser/Thr protein phosphatase calcineurin. Application of PACAPs failed to rescue cartilage formation when the activity of calcineurin was pharmacologically inhibited with cyclosporine A. Moreover, exogenous PACAPs prevented diminishing of cartilage formation and decrease of calcineurin activity during oxidative stress. As an unexpected phenomenon, PACAP 6-38 elicited similar effects to those of PACAP 1-38, although to a different extent. On the basis of the above results, we propose calcineurin as a downstream target of PACAP signalling in differentiating chondrocytes either in normal or pathophysiological conditions. Our observations imply the therapeutical perspective that PACAP can be applied as a natural agent that may have protecting effect during joint inflammation and/or may promote cartilage regeneration during degenerative diseases of articular cartilage.

## Introduction

Originally isolated from ovine hypothalamic extracts, pituitary adenylate cyclase activating polypeptide (PACAP) is a member of the VIP–Secretin–GHRH–Glucagon superfamily [1]. The active neuropeptide comprises 38 amino acid residues (PACAP 1-38); however, a shorter α-amidated form that corresponds to the first 27 residues at the N-terminus (PACAP 1-27) also exists, suggesting that their biologically active region is completely preserved during evolution [1]. PACAP 1-38, the dominant form of the neuropeptide, is widely distributed in the central nervous system (CNS) and is also present in several peripheral tissues such as gonads [2], intestinal system [3], urinary tract [4], as well as in fluid compartments including blood plasma [5] and human milk [6]. In spite of the large variety of tissues containing or releasing PACAP, there are only sporadic data about its function in skeletal elements such as bone [7]–[9]; in particular, its presence in hyaline cartilage has not been reported. PACAP is a multifunctional peptide that can regulate diverse physiological processes such as testicular ageing [10], circadian rhythm [11], brain development [12] or cell migration [13]. PACAP is also regarded as a neurotrophic [14], [15] and anti-inflammatory substance in the CNS [16] and is reported to prevent harmful effects of oxidative stress in various tissues [17]–[19].

So far, three G protein-coupled receptors have been identified on which PACAP can act: PAC1, VPAC1 and VPAC2 [20]. PACAP and VIP bind to VPAC1 and VPAC2 with similar affinity, while PAC1 receptor has approximately 100-fold greater affinity to PACAP [21]. PACAP 6-38, a peptide that lacks the first 5 amino acids from the N-terminus of PACAP 1-38, is a potent pharmacological antagonist of PAC1 receptor [22]. PACAP 6-38 has also been referred to as an antagonist of VPAC2 receptor [23].

Binding of PACAP hormones to their receptors preferentially stimulates adenylate cyclase and increases the concentration of intracellular cAMP. PACAP signalling also utilises other intracellular messengers and pathways including calcium, phospholipase D or diacylglycerol (DAG) and may consequently induce or inhibit the activation of diverse signalling processes that regulate proliferation, gene expression or cytosolic Ca^2+^ concentration of various cell types [24]. The increased level of cAMP can activate protein kinase A (PKA), which may augment CREB phosphorylation *e.g.* in neuronal cells [25].

During inflammatory diseases of articular cartilage, a considerable amount of reactive oxygen species (ROS) is liberated [26]. ROS secretion by chondrocytes in turn may cause structural and functional changes in the extracellular matrix (ECM) of hyaline cartilage [27], [28]. The elevated production of ROS is one of the key factors triggering the induction of apoptosis that leads to chondrocyte loss [29]. The downstream targets of ROS-induced cell responses have been extensively studied in chondrocytes [30], [31] or in synoviocytes [32], but only a few pharmacological compounds, such as thioredoxin and erdosteine, have been identified so far as potent oxidative stress-preventing agents [33]. However, PACAP signalling and its anti-oxidative effects have not been studied during *in vitro* chondrogenesis.

A well-reproducible *in vitro* experimental model for hyaline cartilage differentiation is the chondrifying high density mesenchymal cell culture system (HDC), established from chondroprogenitor cells isolated from limb buds of early chicken embryos in the Hamburger–Hamilton developmental stage 22–24 [34], [35]. In HDC, chondrogenesis occurs spontaneously in six days, and a considerable amount of metachromatic cartilage matrix can be detected by the end of the culturing period. Differentiation of chondrocytes is a complex process. An important initial step of either *in vivo* or *in vitro* chondrogenesis is rapid proliferation [36] and migration of chondroprogenitor cells that bring about precartilaginous cell aggregates. This process occurs during the first two culturing days in HDC, followed by final commitment of chondrogenic cells on day 3 of culturing, which is characterised by activation of genes encoding cartilage-specific ECM macromolecules, *e.g.* type II collagen or aggrecan core protein. In parallel to the intracellular changes, differentiating chondrogenic cells start to secrete a cartilage-specific matrix abundant in sulphated glycosaminoglycans (GAGs) and collagen type II. Chondrocyte differentiation is regulated by several transcription factors; CREB and Sox9 are required for the transcription of mRNAs for collagen type II and aggrecan core protein (reviewed in [37]). Activity of either CREB or Sox9 is triggered or enhanced by transient Ser/Thr phosphorylation; moreover, Sox9 protein expression has a peak on days 2–3 of culturing in HDC [38]. Several protein kinases such as PKA, protein kinase C or MAP kinases are known to phosphorylate both transcription factors [39], [40]. According to our previous findings in chondrogenic cells, the reversibility of this modification is accomplished by PP2A [36] and/or calcineurin (PP2B) [31].

PP2B is a Ser/Thr specific Ca^2+^-calmodulin dependent phosphoprotein phosphatase (PP) consisting of a catalytic subunit and a regulatory subunit, which effectively binds four Ca^2+^ ions on its calcium-binding site, while the catalytic subunit harbours a calmodulin binding region. As rise in free cytosolic Ca^2+^ concentration may result in activation of calcineurin, this PP is an important effector of Ca^2+^-dependent signal transduction pathways [41]. Calcineurin is reported to be a positive regulator of chondrogenesis via the activation of nuclear factor of activated T cells-4 (NFAT4); moreover, it has been found to be a key mediator of oxidative stress-induced signalling in chondrogenic cells [31], [42].

The major goal of the present study was to identify elements and putative role of PACAP signalling in differentiating chondrogenic cells and to investigate the possibility that PACAP exerts chondroprotective effects during oxidative stress. Here we report that chondrogenic cells express components of PACAP signalling and give evidence for a positive role of PACAP in the regulation of *in vitro* chondrogenesis under physiological circumstances and during oxidative stress. We propose calcineurin as a mediator of the chondrogenesis-promoting effect of PACAP-signalling and we prove that this enzyme also plays role in PACAP-related protection of chondrogenesis in oxidative stress. Interestingly, PACAP 6-38 was not found to be acting as an antagonist of PACAP-signalling in our chondrogenesis model.

## Materials and Methods

### 1. Cell culturing

Ross hybrid chicken embryos of Hamburger–Hamilton stages 22–24 were used to establish primary micromass chondrifying mesenchymal cell cultures. Distal parts of the limb buds of embryos were removed and a single-cell suspension of chondrogenic cells at a density of 1.5×10^7^ cells/mL was yielded. Droplets of different volumes of the cell suspension were inoculated into Petri dishes or plates (Orange Scientifique, Braine-l'Alleud, Belgium). Day of inoculation was considered as day 0. Colonies were fed Ham's F12 medium (Sigma, St. Louis, MO, USA), supplemented with 10% foetal calf serum (Lonza Group Ltd, Basel, Switzerland) and were kept at 37°C in the presence of 5% CO_2_ and 80% humidity in a CO_2_ incubator. The medium was changed on every second day. Cultures were maintained for 6 days.

### 2. Administration of PACAP polypeptides and/or H_2_O_2_, CSA on various days of culturing

PACAP 1-38 at 100 nM and 10 µM (stock solution: 100 µM, dissolved in sterile distilled water) was used as agonist of PAC1 receptor; as an antagonist, PACAP 6-38 at 100 nM, 1 µM and 10 µM (stock solution: 10 mM, dissolved in sterile distilled water) was applied continuously from day 1. Synthetic PACAPs were provided by Gábor Tóth, Department of Medical Chemistry, University of Szeged, Faculty of Medicine. To induce oxidative stress, H_2_O_2_ was applied at 4 mM for 20 min on day 3 of culturing. Calcineurin activity was inhibited with 2 µM cyclosporine A (CSA; Sigma; stock solution: 2 mM, dissolved in DMSO) applied continuously from day 1.

### 3. Light microscopical morphology

HDC established from 30 µL droplets of the cell suspension of different experimental groups were cultured on round coverslips (Menzel-Gläser, Menzel GmbH, Braunschweig, Germany) placed into 24-well culture plates. On day 6, cell cultures were fixed in a 4∶1 mixture of absolute ethanol and 40% formaldehyde and stained with 0.1% dimethyl methylene blue (DMMB, Aldrich, Germany) dissolved in 3% acetic acid. Photomicrographs of metachromatic cartilaginous nodules were taken with Spot Advanced camera on a Nikon Eclipse E800 microscope (Nikon Eclipse 800, Tokyo, Japan). The amount of sulphated matrix components was determined with a semiquantitative method by measuring the optical density of extracted toluidine blue (TB, Reanal, Budapest, Hungary) bound to GAGs in mature HDC as it was described previously [43].

### 4. Hyaluronan detection

Cells of HDC were inoculated on the surface of coverslips as described previously. Cells were fixed in Saint-Marie fixative for 1 h. After washing in 70% alcohol, nonspecific binding sites were blocked in phosphate buffered saline with 0.1% Tween 20 (PBST) supplemented with 1% bovine serum albumin (BSA, Amresco, Solon, OH, USA) at 37°C. Hyaluronic acid (HA) was detected by using a biotinylated HA-binding complex in 5 µg/mL concentration (bHABC, kindly provided by R. Tammi and M. Tammi, Department of Anatomy, University of Kuopio, Kuopio, Finland) at 4°C overnight. The reaction was visualised with Streptavidin-Alexa555 (2 µg/mL, Invitrogen Corporation, Carlsbad, CA, USA) for fluorescence microscopy. Cultures were mounted in Vectashield Hard Set mounting medium (Vector Laboratories, Ltd. Peterborough, UK) containing DAPI to visualise the nuclei of cells. Cultures were viewed by fluorescence microscopy (Nikon Eclipse E800). For investigation of proper reactivity, saturation of bHABC with human (Sigma) and Streptomyces (Sigma) derived HA was performed prior to the application of the HA-probe to the cell cultures. Neither signals nor background were observed (data not shown). All images were acquired using constant camera settings to allow comparison of staining intensities.

### 5. Cell proliferation and viability assays

Ham's F12 medium containing 1 µCi/mL ^3^H-thymidine (diluted from methyl-^3^H-thymidine; 185 GBq/mmol, Amersham Biosciences, Budapest, Hungary) was added to wells of special opaque 96-well microtiter plates (Wallac, PerkinElmer Life and Analytical Sciences, Shelton, CT, USA) for 16 h on day 3. For cultures exposed to oxidative stress, this procedure was started promptly after the 20-min-long treatment by H_2_O_2_. Colonies were fixed with ice-cold 5% trichloroacetic acid, air-dried for 1 week and radioactivity was counted by Chameleon liquid scintillation counter (Chameleon, Hidex, Turku, Finland).

For investigation of cellular viability mitochondrial activity was tested with MTT-assay. Cells cultured in 96-well plates were used, 10 µL MTT reagent (3-[4,5-dimethylthiazolyl-2]-2,5-diphenyltetrazolium bromide; 25 mg MTT/5 mL PBS) was pipetted into each well immediately or 16 h after oxidative stress. Cells were incubated for 2 h at 37°C and following addition of 500 µL MTT solubilising solution, absorption of samples was measured at 570 nm (Chameleon, Hidex).

### 6. RT-PCR analysis

Cell cultures were dissolved in Trizol (Applied Biosystems, Foster City, CA, USA), and after the addition of 20% RNase free chloroform samples were centrifuged at 4°C at 10,000×*g* for 15 min. Samples were incubated in 500 µL of RNase-free isopropanol at −20°C for 1 h then total RNA was harvested in RNase free water and stored at −20°C. The assay mixture for reverse transcriptase reaction contained 2 µg RNA, 0.112 µM oligo(dT), 0.5 mM dNTP, 200 units of High Capacity RT (Applied Bio-Systems) in 1× RT buffer. For the sequences of primer pairs and further details of polymerase chain reactions, see Table 1. Amplifications were performed in a thermal cycler (Labnet MultiGene™ 96-well Gradient Thermal Cycler; Labnet International, Edison, NJ, USA) in a final volume of 25 µL (containing 1 µL forward and reverse primers [0.4 µM], 0,5 µL dNTP [200 µM], and 5 units of Promega GoTaq® DNA polymerase in 1× reaction buffer) as follows: 95°C, 2 min, followed by 35 cycles (denaturation, 94°C, 1 min; annealing at optimised temperatures as given in Table 1 for 1 min; extension, 72°C, 90 sec) and then 72°C, 10 min. PCR products were analysed by electrophoresis in 1.2% agarose gel containing ethidium bromide. GAPDH was used as internal control. Optical density of signals was measured by using ImageJ 1.40 g freeware and results were normalised to the optical density of untreated control cultures.

**Table 1:** Nucleotide sequences, amplification sites, GenBank accession numbers, amplimer sizes and PCR reaction conditions for each primer pair are shown.

*Gene*	*Primer*	*Nucleotide sequence (5′→3′)*	*GenBank ID*	*Annealing temperature*	*Amplimer size (bp)*
**Aggrecan core protein**	sense	CAA TGC AGA GTA CAG AGA	**XM_001232949**	54°C	429
		(276–294)			
**(Agr1)**	antisense	TCT GTC TCA CGG ACA CCG			
		(688–704)			
**Chst11**	sense	GGT TTT GAC GGG AAG AGG C	**XM_416316**	56°C	413
		(957–975)			
	antisense	CGT AGT GAA TGT GGC AAG GGT			
		(1349–1369)			
**Collagen II**	sense	GGA CCC AAA GGA CAG ACG G	**NM_204426**	59°C	401
		(1191–1210)			
**(Col2a1)**	antisense	TCG CCA GGA GCA CCA GTT			
		(1573–1591)			
**CREB**	sense	GCT ATT ACG CAA GGA GGA	**NM_204450**	48°C	114
		(725–742)			
	antisense	AAT GGT AGT GCC AGG TTG			
		(821–838)			
**Extl1**	sense	CCA AGG CAG CCA TCG TAG	**NM_004455**	54°C	289
		(1808–1825)			
	antisense	CCT GAT TAT CTT CCC TTC TA			
		(2075–2096)			
**HAS1**	sense	CCT ACG AGG CGG TGG TCT	**NM_001523**	56°C	306
		(1240–1257)			
	antisense	GCA GAG GGA CGT AGT TAG CG			
		(1526–1545)			
**HAS2**	sense	GAG ACG ACA GGC ATC TAA CTA AC	**AF_106940**	52°C	330
		(1497–1519)			
	antisense	AAG ACT TTA TCA GGC CCA CTA A			
		(1805–1826)			
**HAS3**	sense	ATT GCC ACC GTC ATC CAG	**XM_425137**	60.4°C	479
		(826–843)			
	antisense	ACC TCG GCG AAG ACC AAC			
		(1287–1304)			
**NFAT4**	sense	AGC CAG TAT TGA TTG TGC	**NM_173165**	46°C	354
		(1912–1929)			
**(NFATc3)**	antisense	CCTGATTATCTTCCCTTCTA			
		(2246–2265)			
**PAC1**	sense	GTC AGA CAA CCA GGA TTA C	**NM_001098606**	49°C	141
		(435–453)			
**(ADCYAP1R1)**	antisense	TGG ATA AAG TTC CGA GTG			
		(559–575)			
**PKA**	sense	GCA AAG GCT ACA ACA AGG C	**NM_008854**	53°C	280
		(847–865)			
**(Prkaca)**	antisense	ATG GCA ATC CAG TCA ATC G			
		(1109–1126)			
**PP2B**	sense	CTG CTC TGA TGAA CCA ACA GTT	**XM_420664**	54°C	504
		(647–668)			
**(PPP3CA)**	antisense	ACG GCA AGG ACC AGG TAA ACA			
		(1130–1150)			
**Sox9**	sense	CCC CAA CGC CAT CTT CAA	**NM_204281**	54°C	381
		(713–731)			
	antisense	CTG CTG ATG CCG TAG GTA			
		(1075–1093)			
**VPAC1**	sense	GTT CTA TGG CAC GGT CAA	**NM_001097523**	52°C	216
		(376–393)			
**(VIPR1)**	antisense	AGC AAT GTT CGG GTT CTC			
		(573–590)			
**VPAC2**	sense	TCG GAA CTA CAT CCA TCT	**NM_001014970**	48°C	177
		(477–497)			
**(VIPR2)**	antisense	TTT GCC ATA ACA CCA TAC			
		(636–653)			
**Xylt1**	sense	AGC GAT CCA ATT ACT TAC ACC	**XM_414904**	52,5°C	407
		(1103–1123)			
	antisense	GAG AAA CCA ATC CGA CCC			
		(1492–1509)			
**Xylt2**	sense	TCC TGT CCA AAT ACC GAG ATA A	**NM_001001785**	54°C	208
		(1037–1058)			
	antisense	ACC ACG TAC TGC ACG AAG C			
		(1226–1244)			
**GAPDH**	sense	GAG AAC GGG AAA CTT GTC AT	**NM_204305**	54°C	556
		(238–258)			
	antisense	GGC AGG TCA GGT CAA CAA			
		(775–793)			

### 7. Preparation of cell extracts

Cell cultures were washed in physiological NaCl solution and were harvested. After centrifugation, cell pellets were suspended in 100 µL of homogenization buffer containing 50 mM Tris–HCl buffer (pH 7.0), 10 µg/mL Gordox, 10 µg/mL leupeptine, 1 mM phenylmethylsulphonyl-fluoride (PMSF), 5 mM benzamidine, 10 µg/mL trypsin inhibitor as protease inhibitors. Samples were stored at −70°C. Suspensions were sonicated by pulsing burst for 30 sec at 40 A (Cole-Parmer, Illinois, USA). For Western blotting, total cell lysates were used.

### 8. Western blot analysis

Samples for SDS–PAGE were prepared by the addition of fivefold concentrated electrophoresis sample buffer (20 mM Tris–HCl pH 7.4, 0.01% bromophenol blue dissolved in 10% SDS, 100 mM β-mercaptoethanol) to cell lysates to set equal protein concentration in samples, and boiled for 10 min. About 70–80 µg of protein was separated by 7,5% SDS-PAGE gel for detection of PAC1, Sox9, P-Sox9, PKA, PP2B, NFAT4, GAPDH, HAS1, HAS2, HAS3, Aggrecan, Collagen type II, Chst11, CREB and P-CREB. Proteins were transferred electrophoretically to nitrocellulose membranes. After blocking with 5% non-fat dry milk in PBST (containing 0.1% Tween 20), membranes were washed and exposed to the primary antibodies overnight at 4°C. Polyclonal anti-Sox9 antibody (Abcam, Cambridge, UK) in 1∶600, polyclonal anti-P-Sox9 antibody (Sigma) in 1∶800, polyclonal anti-PAC1 antibody (Sigma) in 1∶400, polyclonal anti-PKA antibody (Cell Signaling, Danvers, MA, USA) in 1∶600, polyclonal anti-PP2B antibody (Cell Signaling) in 1∶600, polyclonal anti-NFAT4 antibody (Cell Signaling) in 1∶400, polyclonal anti-HAS1 (Santa Cruz Biotechnology Inc., Santa Cruz, CA, USA) in 1∶200, polyclonal anti-HAS2 (Santa Cruz Biotechnology Inc.) in 1∶200, polyclonal anti-HAS3 (Abcam) in 1∶400, polyclonal anti-Aggrecan (Millipore, Billerica, MA, USA) in 1∶400, monoclonal anti-Collagen type II (Chemicon-Millipore) in 1∶200, polyclonal anti-Chst11 (Sigma) in 1∶400, polyclonal anti-CREB antibody (Millipore) in 1∶800, polyclonal anti-P-CREB antibody (Millipore) in 1∶800 dilution and anti-GAPDH antibody (Abcam) in 1∶1000 were used. After washing for 30 minutes in PBST, membranes were incubated with anti-rabbit IgG (Bio-Rad Laboratories, CA, USA) in 1∶1500, anti-mouse IgG (Bio-Rad Laboratories) in 1∶1500 or anti-goat IgG (Sigma) in 1∶2000 dilution. Signals were detected by enhanced chemiluminescence (Millipore) according to the instructions of the manufacturer. Signals were manually developed on X-ray films. Optical density of Western blot signals was measured by using ImageJ 1.40 g freeware and results were normalised to that of untreated control cultures.

### 9. Calcineurin activity assay

For *in vitro* calcineurin activity assays, cells were harvested in homogenization buffer and suspensions were sonicated by pulsing burst on ice for 30 sec at 40 A (see above). After centrifugation at 10,000×*g* for 10 min at 4°C, supernatants with equal protein concentrations were used for enzyme activity measurements. Activity of calcineurin was assayed by using RII phosphopeptide substrate, and the release of free PO_4_ was detected by a classic malachite green assay (Abcam). Measurements were performed according to the instructions of the manufacturer in three independent experiments.

### 10. Determination of cytosolic free Ca^2+^ concentration

Measurements were performed on day 3 and 16 h later on day 4 on cultures seeded onto 30 mm round coverslips using the calcium-dependent fluorescent dye Fura-2 as described previously [43]. Fura-2-loaded cells were placed on the stage of an inverted fluorescent microscope (Diaphot, Nikon, Kowasaki, Japan) and viewed using a 40× oil immersion objective. Measurements were carried out in Tyrode's salt solution (containing 1.8 mM Ca^2+^; composition: 137 mM NaCl, 5.4 mM KCl, 0.5 mM MgCl_2_, 1.8 mM CaCl_2_, 11.8 mM HEPES, 1 g/L glucose; pH 7.4) in a perfusion chamber using a dual wavelength monochromator equipment (DeltaScan, Photon Technologies International, Lawrenceville, KY, USA) at room temperature. Excitation wavelength was altered between 340 and 380 nm at 50 Hz and emission wavelength was detected at 510 nm. Data acquisition frequency was 10 Hz. Ratios of emitted fluorescence intensities (detected at alternating excitation wavelengths; F_340_/F_380_) were measured as previously described [43]. Cytosolic Ca^2+^ concentration was determined on day 3 directly after PACAP and/or H_2_O_2_ administration and 16 h later on day 4. Intracellular Ca^2+^ levels in cells of untreated control cultures were determined in 3 independent experiments, measuring 30 cells in each case.

### 11. Statistical analysis

All data are representative of at least three independent experiments. Where applicable, data are expressed as mean ± SEM. Statistical analysis was performed by One Way ANOVA test combined with post hoc tests. Where ANOVA reported significant differences among the groups (P<0.05) a post hoc test (multiple comparison *versus* control group, Dunnett's method) was used to isolate the groups that differed from the control group at p<0.05. The respective control group was the untreated control when comparison was made among control, H_2_O_2_-treated, PACAP 1-38-treated and PACAP 6-38-treated groups, whereas the H_2_O_2_-treated cultures were used as control in the post hoc test when comparison was made among H_2_O_2_-treated, H_2_O_2_+PACAP 1-38-treated and H_2_O_2_+PACAP 6-38-treated samples. Statistical analysis and comparison of Western blot and PCR results can be seen in Figures S3A, S3B, S3C, S3D, S3E, S3F, S3G, S3H and S3I.

## Results

### 1. Identification of PACAP and its receptors in chondrifying cells

**Figure 1:**
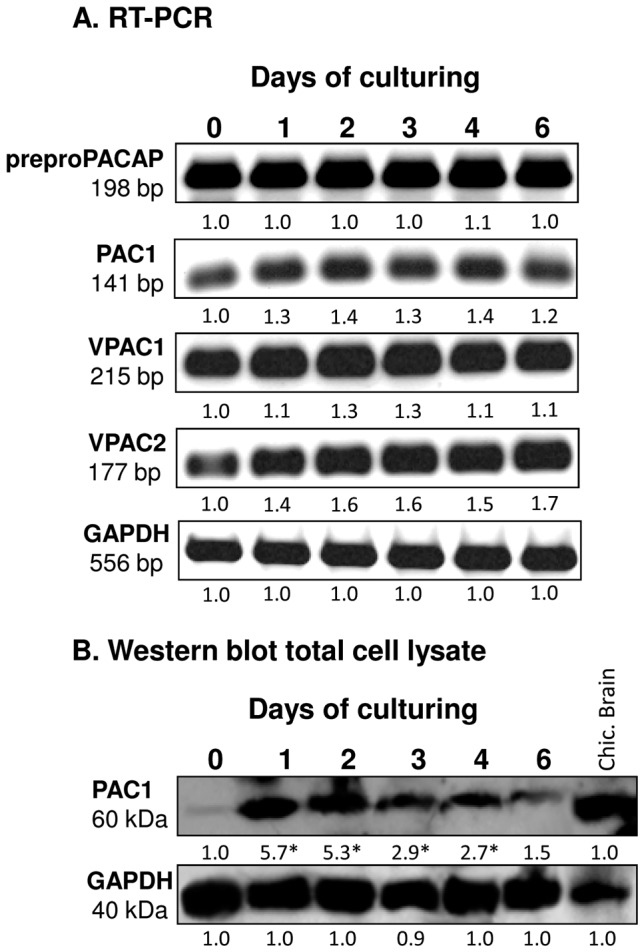
Expression of preproPACAP and PACAP receptors in chondrifying micromass cultures. For RT-PCR (A) and Western blot (B) reactions, GAPDH was used as control. Optical density of signals was measured and results were normalised to the optical density of 0-day-old cultures. Representative data of 3 independent experiments. For panels (A) and (B), numbers below signals represent integrated densities of signals determined by ImageJ software.

mRNA expressions of preproPACAP, as well as those of PACAP receptors, *i.e.* PAC1, VPAC1 and VPAC2 were detected throughout the 6-day-long culturing period in chicken HDC (Figure 1A, Figure S3A). As PACAP has a 100-fold higher binding affinity to the PAC1 receptor compared to VPAC receptors [21], we only investigated the protein expression of PAC1 in this study. Protein expression of the PAC1 receptor showed a peak-like pattern with a five-fold elevation on days 1 and 2 (when chondrogenic cells predominantly differentiate to chondroblasts) compared to day 0 (Figure 1B, Figure S3B). Chicken brain lysate was used as positive control.

### 2. Administration of PACAP proteins augments cartilage formation

**Figure 2:**
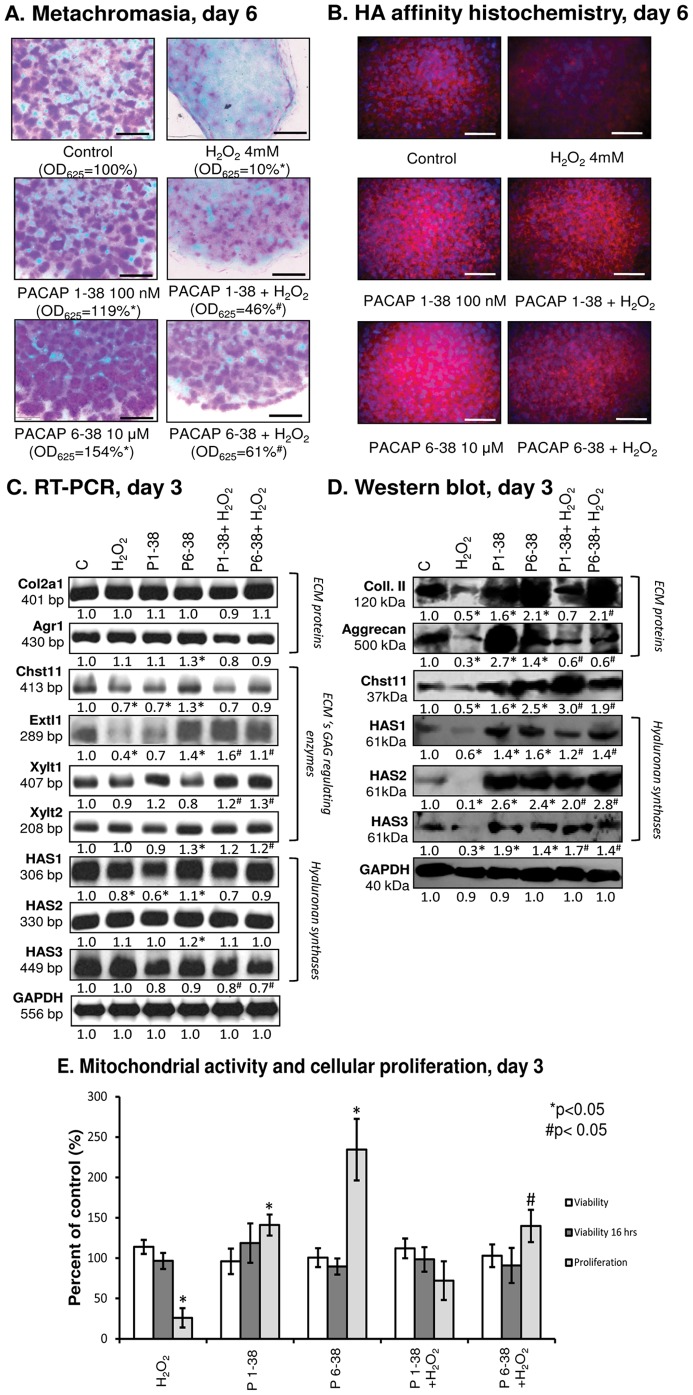
Effects of PACAP and/or H_2_O_2_ on matrix production, mitochondrial activity and proliferation rate of HDC. PACAP 1-38 at 100 nM, PACAP 6-38 at 10 µM were applied continuously from day 1. (A) Metachromatic cartilage areas in 6-day-old cultures were visualized with DMMB dissolved in 3% acetic acid. Metachromatic (purple) structures represent cartilaginous nodules formed by many cells and cartilage matrix rich in sulphated GAGs and PGs. Original magnification was 4×. Scale bar, 500 µm. Optical density (OD_625_) was determined in samples containing TB extracted with 8% HCl dissolved in absolute ethanol. (B) HA production of HDC was determined by HA affinity reaction. Red fluorescence represents HA in ECM of a representative cartilage nodule on day 6 of culturing. Original magnification was 40×. Scale bar, 20 µm. (C) RNA expression of aggrecan core protein *(Agr1)*, collagen type II *(Col2a1)*, chondroitin 4-O-sulfotransferase 1 *(Chst111)*, 1,4-N-acetylglucosaminyltransferase *(Extl1)*, xylosyltransferase 1 and 2 (*Xylt1* and *Xylt2*), and HAS1, HAS2 and HAS3 on day 3. GAPDH was used as a control. (D) Protein expression of aggrecan core protein, collagen type II, chondroitin 4-O-sulfotransferase 1 (Chstl 11), HAS1, HAS2 and HAS3 on day 3. GAPDH was used as a control. (E) Effects of PACAP and/or H_2_O_2_ administration on mitochondrial metabolic activity and cellular proliferation in HDC on culturing day 3. Asterisks indicate significant (*P<0.05) alteration of cell proliferation or matrix production as compared to the respective control; hash signs indicate significant (^#^ *P*<0.05) difference in proliferation rate and cartilage formation as compared to the respective H_2_O_2_ treatment. For panels (C) and (D) numbers below signals represent integrated densities of signals determined by ImageJ software. Representative data of 3 independent experiments are shown. (C, control; P1-38, PACAP 1-38; P6-38, PACAP 6-38).

Continuous application of PACAP 1-38 at 100 nM or 10 µM from day 1 of culturing equally elevated cartilage matrix production by 20% compared to the control as revealed by DMMB and TB staining procedures (Figure 2A, Figure S1). Surprisingly, when PACAP 6-38, a potent antagonist of PAC1 and VPAC2 receptors [22] was applied, we observed concentration-dependent changes in metachromatic cartilage ECM formation: while no change was caused by either 100 nM or 1 µM PACAP 6-38 (Figure S1), a pronounced (1.5-fold) elevation of metachromatic matrix production in HDC was measured when applied at 10 µM (Figure 2A). Metachromatic staining allows approximation of the amount of sulphated GAGs and PGs in the cartilage matrix and is a good indicator of the effectiveness of chondrogenesis. However, changes in the amount of the non-sulphated GAG hyaluronan (HA) cannot be evaluated with this method. In order to monitor effects of PACAPs on the presence of this ECM component HA affinity assays were performed. Similar to what has been observed following metachromatic staining procedures, applications of PACAPs resulted in more intense fluorescent signals for HA. In our multicellular cell cultures, fine details of the localization of HA cannot be determined with this method, but an overall increase of the HA-amount was well ascertainable (Figure 2B).

As far as the expression of cartilage-specific ECM components is concerned, the mRNA level of *Col2a1* (collagen type II) remained steady under the effect of both PACAPs; in contrast, *Agr1* (aggrecan core protein) was found to be slightly upregulated in the presence of PACAP 6-38, while no change was detected by PACAP 1-38 treatment (Figure 2C, Figure S3C). Sulphated GAGs and PGs in cartilage ECM are synthesised and assembled by several enzymes such as 1,4-N-acetylglucosaminyltransferase (*Extl1*), chondroitin 4-O-sulfotransferase 1 (*Chst11*), xylosyltransferase 1 and 2 (*Xylt1*, *Xylt2*); therefore, the mRNA expressions of these enzymes were also investigated. As an obvious elevation of non-sulphated HA in cartilage ECM also was observed and HA acts as a scaffold to which aggrecan monomers are bound to form large aggregates, mRNA expression patterns of HA synthase enzymes (HAS1, HAS2, HAS3) were also monitored. Under the effect of PACAP 6-38, we detected moderate elevations in the mRNA expression of *Extl1*, *Chst11*, *Xylt2* and HAS2, while administration of PACAP 1-38 slightly decreased the expression of these enzymes, except for *Xylt1* and HAS2, whose expression was not significantly altered (Figure 2C, Figure S3C).

Investigation of protein expression of aggrecan and collagen type II resulted in a pattern comparable to that of metachromatic matrix production. Administration of PACAP 1-38 elevated collagen type II expression and had an even stronger stimulatory effect on aggrecan expression (Figure 2D, Figure S3D). Moreover, the presence of PACAP 6-38 had a well-pronounced protein expression-increasing effect on collagen type II and a smaller but significant elevation of aggrecan expression was also detected (Figure 2D). Enhanced Chst11 protein expression was shown in the presence of the neuropeptides indicating augmented GAG accumulation in the ECM of HDC. As a result of PACAP treatments, the elevated protein expression of all three HAS isotypes showed a good correlation with the results of HA-affinity histochemistry (Figure 2B and D).

The increased amount of cartilage matrix can also be a consequence of higher number of chondrogenic cells as a result of more intense proliferation. Although the mitochondrial activity of differentiating cells was not altered either on day 3 or 4 by PACAP treatments as revealed by MTT assays, administration of both PACAPs caused a significant increase in proliferation rate (Figure 2E).

### 3. PACAP 1-38 and 6-38 exert protective function during cartilage formation under oxidative stress

Oxidative stress was induced by the application of H_2_O_2_ at 4 mM for 20 min on the day of final commitment of chondrogenic cells (day 3). In accordance with our previously published results [31], ROS diminished the formation of metachromatic cartilage nodules (Figure 2A) without causing any significant changes in viability (Figure 2E) or apoptotic or necrotic processes of the differentiating cells (Figure S2). When H_2_O_2_ was added to cultures previously treated with PACAP 1-38 or PACAP 6-38, metachromatic cartilage matrix formation was at least partially rescued (40% or 60% of untreated controls, respectively; Figure 2A). ROS also decreased the HA content of ECM, but the application of PACAP neuropeptides reduced the effect of oxidative stress (Figure 2B). The mRNA expressions of *Col2a1* and *Agr1* were not altered by the presence of ROS (Figure 2C, Figure S3C). In contrast, the protein levels of collagen type II and aggrecan were significantly decreased in the presence of H_2_O_2_, which was prevented by both PACAP 1-38 and PACAP 6-38 (Figure 2D, Figure S3D). The mRNA expressions of *Extl1* and *Chst11* decreased under oxidative stress, which was prevented by pre-treatment with PACAPs (Figure 2C, Figure S3C). Furthermore, the decrease in protein expression of Chst11 during oxidative stress was prevented by PACAPs; moreover, it was intensified compared to PACAP treated cultures (Figure 2D, Figure S3D). Administration of PACAPs during oxidative stress resulted in a slight elevation in the mRNA expression of *Xylt1* and *Xylt2*. Expressions of HAS1 and HAS2 were not altered significantly by either H_2_O_2_ or PACAPs; on the contrary, the mRNA expression of HAS3 significantly decreased under combined treatment with PACAPs and ROS (Figure 2C, Figure S3C). Protein levels of all HAS isoforms decreased under the effect of oxidative stress, which was rescued by PACAP neuropeptides (Figure 2D, Figure S3D).

Next, we looked at how oxidative stress modulated cellular proliferation of HDC and whether pretreatment with PACAPs could attenuate these effects. 20-min-long treatment with H_2_O_2_ on day 3 of culturing abrogated cellular proliferation, which was not rescued when cultures were pretreated with PACAP 1-38 (Figure 2E). Interestingly, however, pretreatment with the PAC1-antagonist PACAP 6-38 had a significant protective effect against the proliferation inhibition caused by oxidative stress (Figure 2E).

### 4. PACAP neuropeptides activate PKA, CREB and Sox9 in chondrogenic cells

**Figure 3:**
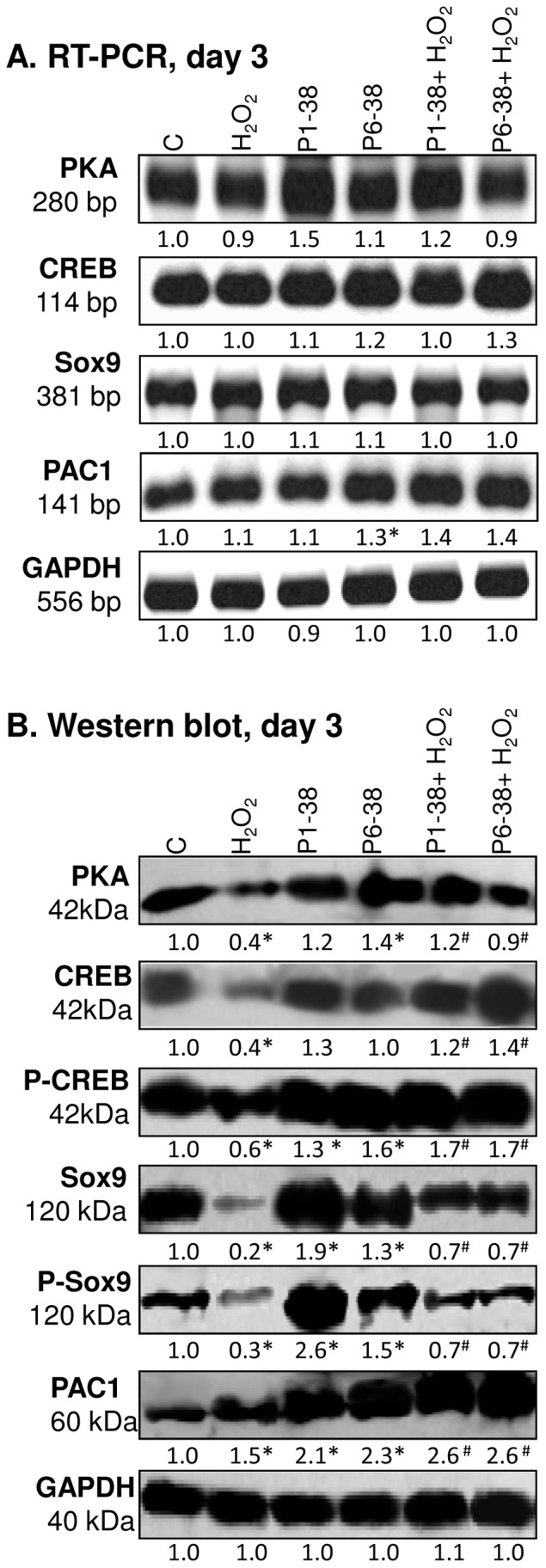
PACAP and oxidative stress influence PKA, CREB, Sox9 and PAC1 receptor expression in chondrogenic cells. PACAP 1-38 at 100 nM, PACAP 6-38 at 10 µM were administrated continuously from day 1. mRNA (A) and protein (B) expression of PKA, CREB, P-CREB, Sox9, P-Sox9 and PAC1 in cells of chondrifying micromass cultures on day 3 of culturing. For RT-PCR and Western blot reactions, GAPDH was used as internal control. Optical density of signals was measured and results were normalised to the optical density of internal controls. For panels (A) and (B) numbers below signals represent integrated densities of signals determined by ImageJ software. Asterisks indicate significant (*P<0.05) alteration of expression as compared to the respective control; hash signs indicate significant (^#^ *P*<0.05) difference in expression as compared to the respective H_2_O_2_ treatment. Representative data of 3 independent experiments. (C, control; P1-38, PACAP 1-38; P6-38, PACAP 6-38).

The “canonical” PACAP-signalling pathway involves the activation of G-protein coupled PAC1 receptor and subsequent elevation of intracellular cAMP concentration, which may lead to the induction of downstream signalling cascades such as PKA activation [44]. PKA is known to phosphorylate CREB and Sox9 transcription factors, which in turn can translocate into the nucleus and may activate the expression of cartilage-specific ECM molecules (*e.g.* collagen type II and/or core protein of aggrecan) in chondrogenic cells [39], [45]. In our experiments, PACAPs only slightly influenced both the mRNA and the protein expression of PKA; however, they rescued PKA protein expression after treatment with H_2_O_2_ (Figure 3A–B, Figure S3E–F).

Addition of PACAP neuropeptides to HDC did not alter significantly the mRNA and protein expression of CREB. The elevation of CREB protein expression was more pronounced during simultaneous application of PACAPs and H_2_O_2_. Although oxidative stress alone significantly decreased both protein expression and phosphorylation level of CREB, the neuropeptides rescued the phosphorylation-decreasing effect of ROS (Figure 3B, Figure S3F).

Neither PACAP 1-38 nor PACAP 6-38 affected the mRNA expression of *Sox9* (Figure 3A). Nevertheless, both neuropeptides markedly elevated its protein expression (Figure 3B, Figure S3E). Phosphorylation of Sox9 on Ser 211 residue by PKA conveys increased transcriptional activity [39]; we observed that the level of P-Sox9 became elevated in the presence of both PACAPs (Figure 3B). Moreover, the reduced expression and phosphorylation of Sox9 protein by oxidative stress was compensated by the administration of PACAPs (Figure 3B, Figure S3F). The protein expression of PAC1 receptor was significantly elevated under oxidative stress and this phenomenon became more pronounced in the simultaneous presence of PACAP neuropeptides (Figure 3B, Figure S3F).

### 5. Calcineurin is a downstream target of PACAP-signalling during chondrogenesis

**Figure 4:**
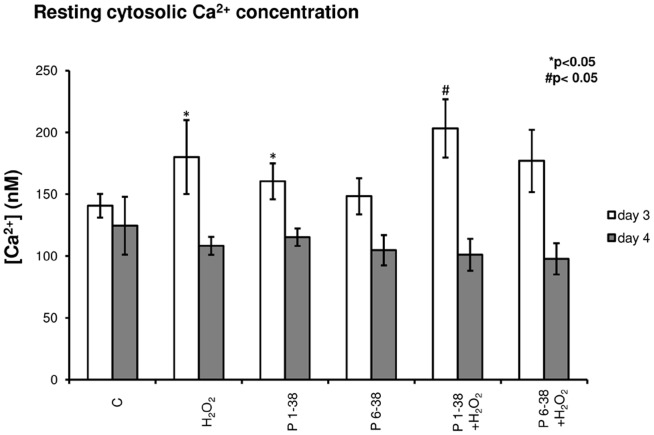
Administration of PACAPs and/or H_2_O_2_ influences Ca^2+^ homeostasis of chondrogenic cells. PACAP 1-38 at 100 nM, PACAP 6-38 at 10 µM were administrated continuously from day 1. Basal cytosolic Ca^2+^ concentration in Fura-2-loaded cells of HDC on days 3 and 4 of culturing. Measurements were carried out in untreated control cultures, during PACAP treatment or directly after oxidative stress. Data shown are mean values of 30 cells in each experimental group. Asterisks indicate significant (*P<0.05) alterations of cytosolic Ca^2+^ concentration as compared to the respective control; hash signs indicate significant (^#^ *P*<0.05) difference in cytosolic Ca^2+^ concentration as compared to the respective H_2_O_2_ treatment. Shown are representative results of 3 independent experiments. (C, control; P1-38, PACAP 1-38; P6-38, PACAP 6-38).

**Figure 5:**
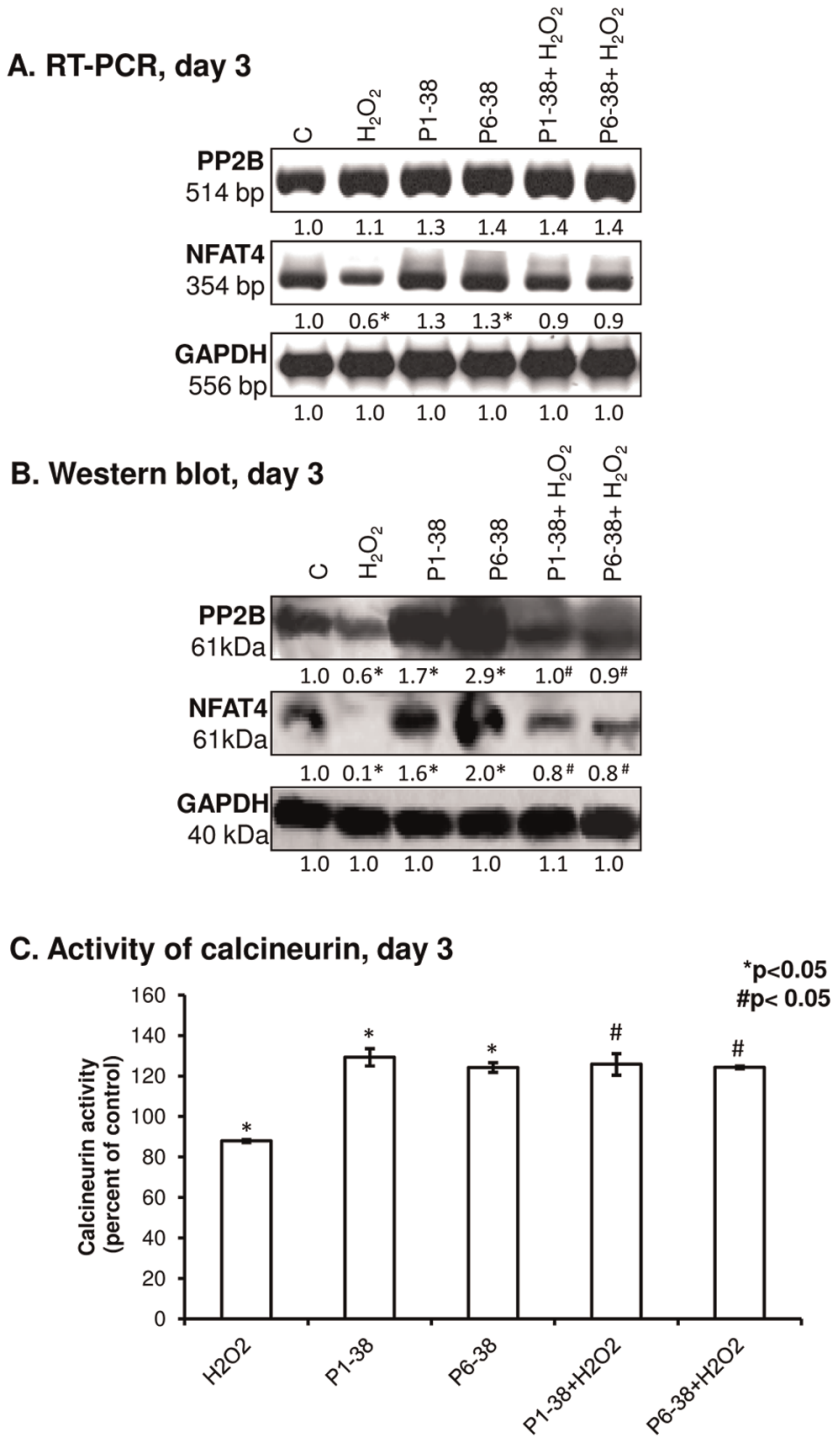
Effects of PACAPs and/or oxidative stress on NFAT4, calcineurin expression and activity in chondrifying cells. PACAP 1-38 at 100 nM, PACAP 6-38 at 10 µM were applied continuously from day 1. (A) mRNA and (B) protein expression of PP2B and NFAT4 in HDC on day 3 of culturing. GAPDH was used as a control. Numbers below to the signals represent integrated densities of signals determined by ImageJ software. (C) Enzyme activity of calcineurin in chondrifying cells on day 3. Asterisks indicate significant (*P<0.05) decrease of calcineurin activity and expression as compared to the respective control; hash signs represent significant (^#^ *P*<0.05) decrease of calcineurin activity and expression as compared to the respective H_2_O_2_ treatment. Representative data of 3 independent experiments. (C, control; P1-38, PACAP 1-38; P6-38, PACAP 6-38).

**Figure 6:**
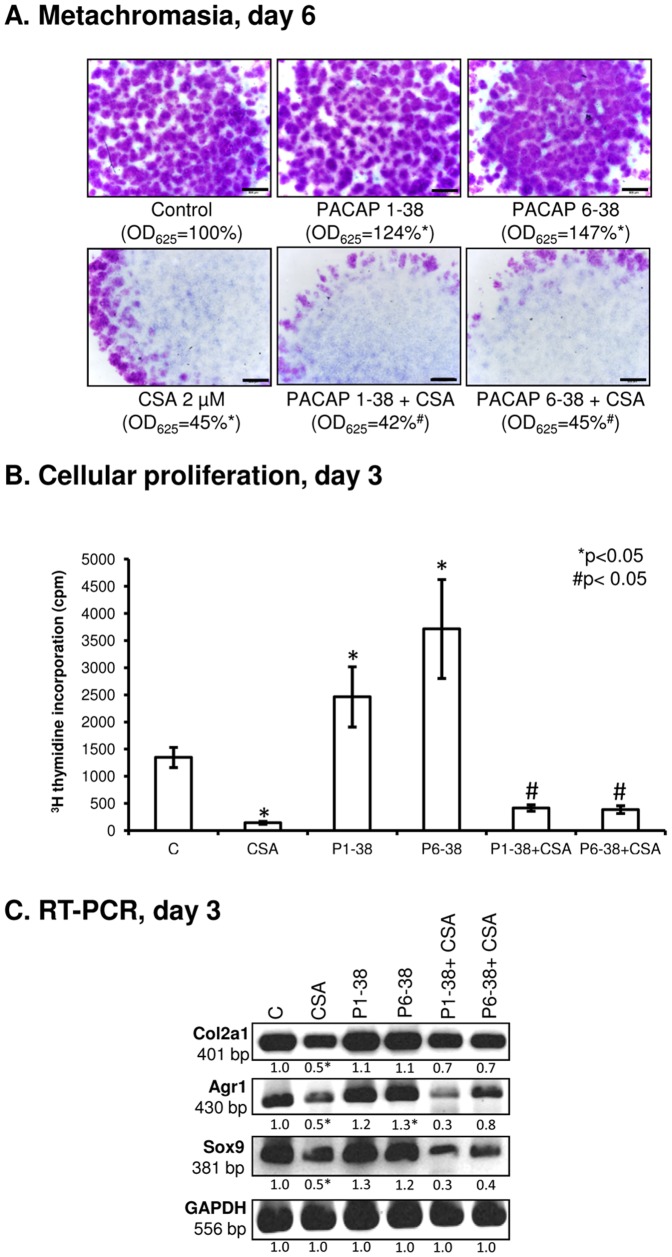
Effects of PACAPs on cartilage formation of HDC when calcineurin was inhibited with cyclosporine A (CSA). CSA at 2 µM was administered continuously from day 1 of culturing with or without PACAPs. (A) Metachromatic cartilage areas in 6-day-old cultures were visualized with DMMB dissolved in 3% acetic acid. Metachromatic (purple) areas represent cartilaginous nodules formed by many cells and cartilage matrix rich in sulphated GAGs and PGs. Original magnification was 4×. Scale bar, 500 µm. Optical density (OD_625_) was determined in samples containing TB extracted with 8% HCl dissolved in absolute ethanol. (E) Effects of PACAP and/or CSA on cellular proliferation in HDC on culturing day 3. (C) RNA expression of collagen type II *(Col2a1)*, aggrecan core protein *(Agr1)* and Sox9 on day 3. GAPDH was used as a control. Representative data of 3 independent experiments are shown. Asterisks indicate significant (*P<0.05) alteration of matrix production, proliferation and mRNA expression as compared to the respective control; hash signs indicate significant (^#^ *P*<0.05) difference in cartilage formation, proliferation and mRNA expression as compared to the respective CSA treatment. (C, control; P1-38, PACAP 1-38; P6-38, PACAP 6-38).

PACAP signalling via PAC1 receptor activation can modulate intracellular Ca2+ concentration [44]. In our experiments, significant elevation of free cytosolic Ca2+ concentration was measured in PACAP 1-38 treated cells either in normal conditions or in oxidative stress, while PACAP 6-38 failed to cause any significant alteration of this parameter (Figure 4). Nonetheless, the above effects proved to be only temporary as Ca2+ levels in all experimental groups returned to control levels when measured 16 h after treatments (Figure 4). It is worth mentioning that chondrogenic cells in HDC are very sensitive to alterations of resting cytosolic free Ca2+ concentration; elevation over a certain threshold inhibits cartilage formation, while smaller increase exerts chondrogenesis-promoting effect [43]. The Ser/Thr phosphoprotein phosphatase calcineurin is amongst the signalling molecules that respond to changes of intracellular Ca2+ concentration. Calcineurin is reported as a positive regulator of chondrogenesis and is also involved in signalling mechanisms evoked by oxidative stress in chondrogenic cells [31]. Expression of calcineurin responded to treatment with PACAPs: although the mRNA expression was not significantly altered (Figure 5A, Figure S3G), protein expression of calcineurin was almost doubled by PACAP 1-38 and a more pronounced, nearly 3-fold stronger signal was detected in the presence of PACAP 6-38 (Figure 5B, Figure S3H). Oxidative stress caused a significant decrease in the expression of calcineurin, and application of PACAPs prevented this effect (Figure 5B, Figure S3H). Activity of calcineurin was elevated by both PACAPs (Figure 5C), while oxidative stress lowered this parameter and administration of the neuropeptides exerted protective effect against this inhibition (Figure 5C). One of the downstream targets of PP2B is NFAT4. Although mRNA expression of this transcription factor showed a significant increase evoked only by PACAP 6-38, significant elevations of NFAT4 protein expression were detected after administration of both PACAPs (Figure 5A–B, Figure S3G–H). Moreover, both neuropeptides exerted a protecting effect against the negative influence of H2O2 on NFAT4 expression (Figure 5A–B). Further scrutinising the role of calcineurin in PACAP signalling we inhibited the activity of calcineurin by CSA, a pharmacological inhibitor of this phosphatase. Following application of CSA, a dramatic decrease in the metachromatic staining of the cell cultures was visible as a result of the chondro-inhibitory effect of this pharmacon and PACAPs failed to rescue cartilage formation under this condition (Figure 6A). Administration of CSA decreased the proliferation rate of chondrogenic cells. The presence of PACAP neuropeptides attenuated the antiproliferative effect of calcineurin inhibition, but proliferation rate still remained very low compared with controls or cultures treated with CSA (Figure 6B). The mRNA expressions of Agr1, Col2a1 and Sox9 were decreased after CSA administration, and PACAPs failed to compensate this effect of the inhibition of calcineurin activity (Figure 6C, Figure S3I).

## Discussion

The presence and functions of PACAP neuropeptides are broadly investigated in various peripheral tissues such as kidney, liver and testis, but little is known about its distribution in cartilage [9], [46]. Since PACAP is known as an important protective compound during various cellular stress responses [4], [14], [16], [47], [48], it could be intriguing whether it influences noxious effects of oxidative stress during cartilage formation or regeneration. Quite recently, migratory chondroprogenitor cells, which may offer regeneration of the damaged tissue to a certain extent, have been isolated from osteoarthritic human articular cartilage [49]. Therefore, any endogenous mechanism to enhance the chondrogenic potential of this unique cell population may have high impact on the healing process.

Here we report the presence and function of the PACAP-signalling toolkit in differentiating chicken chondrocytes and provide evidence for a positive regulatory role of this neuropeptide on chondrogenesis *in vitro*. To our best knowledge, this report is the first to demonstrate the presence of preproPACAP mRNA in chondrogenic cells. We were also able to show that mRNAs of all PACAP receptors are expressed by differentiating chondrocytes. In other skeletal elements such as bone, the expression of PACAP receptors (PAC1, VPAC1 and VPAC2) has already been published [50]. Activation of PACAP receptors has also been investigated in an osteoblast cell line [51]. Of the receptors where PACAP may act as a ligand, PAC1 exhibits the highest affinity to PACAP [21]; therefore, we confirmed the protein expression of this receptor in differentiating chondrocytes. Similar to several other plasma membrane-related signalling molecules involved in the regulation of chondrogenesis in HDC such as the P2X_4_ purinoreceptor [52] or the voltage gated potassium channel K_V_1.1 [53], protein expression of PAC1 receptor also showed a peak on day 3 of culturing, when final commitment of chondrogenic cells occurs in HDC. This finding indicated that PACAP signalling might have a role in this step of chondrogenesis. This idea was supported by reports on a differentiation-promoting effect of PACAP 1-38 in neuronal stem cell development [12], [54] or in the formation of the reproductive system [55]. Indeed, addition of the PAC1 agonist PACAP 1-38 into the medium of chondrifying cell cultures enhanced cartilage formation. Although PACAP 6-38 has been referred to as an antagonist [56] of PAC1/VPAC2 receptors, when administrated at 10 µM a more pronounced cartilage formation-promoting effect was observed compared to PACAP 1-38 applied at the concentration of 100 nM or 10 µM. The “inverse” effect of PACAP 6-38; that is, it acts as an agonist, rather than an antagonist has also been observed by others; there is evidence that it can also act as a PACAP receptor agonist in distantly related tissues such as sensory nerve terminals, glial cells and cytotrophoblasts [57]–[58].

In spite of the pronounced elevation in the amount of cartilage matrix under the effect of PACAPs, mRNA expression level of aggrecan core protein only exhibited a slight change in the presence of PACAP 6-38; moreover, mRNA expressions of matrix-synthesizing enzymes were also altered by PACAP 6-38 only. Surprisingly, the application of PACAP 1-38 did not significantly modulate the mRNA expressions of any of the enzymes mentioned above. In contrast to the protein expression of ECM components such as aggrecan, collagen type II expression was found to be significantly elevated in the presence of both neuropeptides, and similar results were observed for extracellular HA accumulation and HAS enzyme expression patterns. Similarly to our data, others also failed to demonstrate any striking effects of PACAP treatments on gene expression levels along with considerable changes in protein levels and/or phosphorylation of the same molecules: Moody *et al.* reported that the activation of PAC1 receptor with addition of PACAP regulated the tyrosine phosphorylation of FAK rather than significantly influence mRNA expression of these molecules [59]. Furthermore, it has also been shown that PACAP can modulate the expression levels of 120 independent proteins in megakaryocytes without interfering with mRNA expression levels [60]. When we looked at the expression of Sox9, the master transcription factor of chondrogenesis [37], we failed to detect any significant change in its mRNA level again. Nonetheless, Sox9 protein expression was elevated by either PACAP 1-38 or PACAP 6-38, and a similar increase in Sox9 phosphorylation status was also found. These observations raise the possibility that PACAPs augment chondrogenesis and stimulate matrix production *via* influencing translation or protein degradation, rather than modulating transcriptional events.

The first steps of proper cartilage differentiation are also determined by rapid increase in cell number; therefore, the proliferation rate of chondrogenic cells was also investigated. We detected a highly elevated proliferation rate under the effect of PACAPs, which may have significant contribution to the augmented cartilage formation without significant upregulation in transcription of genes encoding matrix molecules and/or their synthesizing enzymes. Nonetheless, the effect of PACAP on cell proliferation is cell type and tissue dependent, since it is known to induce cell division of smooth muscle cells in blood vessels [61] or astrocytes [12], but inhibited the proliferation of retinal progenitor cells [62] and certain tumour cells [63].

The efficient protective role of PACAP in various cellular stress situations has also been widely demonstrated [14], [16], [47]. During inflammatory processes of joints, the accumulation of ROS around and probably within articular cartilage can negatively influence the normal composition of the ECM and may induce apoptosis of chondrocytes *in vivo*. In a good correlation with this, our group has reported a severe reduction of *in vitro* cartilage formation during H_2_O_2_-induced oxidative stress [31]. Among the pleiotropic effects of PACAP it is well demonstrated that it can prevent the apoptotic effect of ROS in astrocytes [19]. The trophic function of PACAP has also been shown in various peripheral tissues such as epithelial cells of kidney [18], endothelial cells [48] and cardiomyocytes [64]. In HDC, we applied 4 mM H_2_O_2_ to evoke oxidative stress during chondrogenesis. This concentration was not cytotoxic [31], but diminished cartilage formation. Pre-treatment with PACAPs partially rescued cartilage formation in H_2_O_2_-treated HDC. This chondro-protective effect of PACAPs during oxidative stress seemed multifactorial: pretreatment with PACAPs rescued Sox9 protein expression and phosphorylation, prevented reduction of HA synthesis and/or aggrecan and collagen type II expression and it also compensated the antiproliferative effect of oxidative stress. Interestingly, PACAP 6-38 exerted an even more pronounced protective effect. As several structural variants of PAC1 receptor with different downstream effectors have been described [65]–[67], the above-described broad spectrum of protecting effect of PACAPs might be the consequence of the fact that distinct downstream signal transduction pathways became activated by the two different neuropeptides *via* binding to different isoforms of PAC1 receptor.

Binding of PACAP to PAC1 receptor generally leads to an increase of intracellular cAMP concentration that triggers the activation of PKA, PKC or MAPK pathways [44]. In the current study, PACAP neuropeptides, at least partly, exerted their effects by acting via the “canonical” PKA-related signal transduction pathway in chondrogenic cells: their administration to the cell cultures significantly elevated the protein expression of PKA. Major downstream targets of the PKA pathway in chondrogenic cells can be the transcription factors Sox9 [39] or CREB [68]. Sox9 can establish a direct interaction with CREB [45], implying the possibility of their parallel activation during chondrogenesis. Indeed, the protein expression and the ratio of the active phosphorylated form of both CREB and Sox9 were increased under the effect of PACAPs. The applied oxidative stress regime decreased the protein expression of PKA, CREB, Sox9 and lowered the phosphorylation level of both transcription factors. The increased protein expression of PAC1 receptor is an interesting phenomenon; however, similar results were observed in rat carotid body under hypoxic conditions [69] or in global brain ischemia in mice [70]; it may be a general cellular response to allow an enhanced PACAP signalling in order to obtain a more efficient cellular protection.

Calcineurin is a positive modulator of chondrogenesis and is a target of oxidative stress in HDC [31], [42]. Our laboratory has demonstrated that pharmacological inhibition of calcineurin with CSA diminished cartilage formation, although the overexpression of the catalytic subunit of this phosphatase also decreased cartilage formation [71], suggesting the importance of the maintenance of calcineurin activity between precisely set levels during chondrogenesis. We have also published that the activity of calcineurin significantly decreased during oxidative stress in a ROS-dose dependent manner and played role in the inhibition of cartilage formation [31]. As PAC1 receptor can regulate cytosolic Ca^2+^ concentration *via* elevation of cAMP concentration [72], we also aimed to explore whether PACAP exerted any effects on the activity of calcineurin in chondrogenic cells. We found that intracellular Ca^2+^ concentration of chondrogenic cells was influenced by these neuropeptides. In line with this, both protein expression and activity of calcineurin markedly increased in the presence of PACAPs. Moreover, PACAPs were able to prevent the PP2B activity-reducing effect of oxidative stress. One of the downstream targets of PP2B can be NFAT4, which promotes chondrogenesis *via* the induction of BMP signalling pathways [73]. Indeed, the positive effect of PACAP on calcineurin activity in chondrogenesis was further supported by the elevated NFAT4 protein expression in HDC, which can partly be responsible for the enhanced cartilage formation. The above results suggest that PACAPs influence multiple signalling pathways in chondrogenic cells: the canonical PKA-pathway and the calcineurin–NFAT–BMP axis can both play role. The significance of the latter signalling pathway was underlined by our observation that pharmacological inhibition of calcineurin activity by CSA diminished cartilage formation [31] and application of PACAPs failed to rescue chondrogenesis in this experimental condition. Furthermore, the application of PACAP neuropeptides was not able to prevent either the severe impairment of cell proliferation or the mRNA expression of cartilage specific molecules when calcineurin was inhibited with CSA. These data further support our concept that calcineurin is a downstream target of PACAP signalling. Indeed, it has been demonstrated the PACAP addition exerted nephroprotective effect during CSA administration in kidneys of mice [74].

Our finding that both PACAP-treatments and oxidative stress resulted in elevated cytosolic Ca^2+^-concentration further complicates the picture. We have reported that during the course of chondrogenesis there was a characteristic pattern in cytosolic Ca^2+^-concentration in chondrogenic cells with a peak at day 3, which pattern was indispensable to proper cartilage formation [43]. Ca^2+^-concentration detected in 3-day-old cells of HDC treated either with PACAP 1-38 or H_2_O_2_ was higher (approximately 160 and 180 µM, respectively) than the precisely regulated Ca^2+^-level of control cells (around 140 µM). In contrast, the administration of PACAP 6-38 did not significantly alter cytosolic Ca^2+^ concentration of chondrogenic cells as it was also demonstrated by others in chromaffin cells [75]. We suppose that H_2_O_2_ treatments inhibited cartilage formation partly via raising intracellular Ca^2+^ concentration over a certain threshold, while PACAPs, especially PACAP 6-38, by modestly lowering Ca^2+^ concentration, may play a balancing role in the regulation of this cellular parameter. This might be one of the mechanisms by which PACAPs rescued or stimulated cartilage formation in this model. Furthermore, the elevated intracellular Ca^2+^ concentration increased the activity of calcineurin following PACAP treatments compared to controls, supporting the hypothesis that a certain range of the elevated Ca^2+^ concentration has positive effects on Ca^2+^-dependent signalling pathways, and sustaining the resting intracellular Ca^2+^ concentration in a tolerable range during oxidative stress by PACAPs may prevent the harmful effects of oxidative stress on signalling pathways of chondrogenic cells.

In summary, our results demonstrate for the first time that chondrogenic cells express molecular elements of PACAP signalling during cartilage differentiation and PACAPs have a chondrogenesis-promoting effect. Moreover, PACAPs rescued cartilage formation during H_2_O_2_-induced oxidative stress, which raises the possibility of the application of PACAPs as a chondroprotecting/regeneration stimulating substance during inflammatory joint diseases. Our findings suggest that PACAPs exert their effects *via* complex signalling mechanisms with considerable cross-talk and we propose that the Ca^2+^-calmodulin dependent PP calcineurin is one of the downstream targets of PACAPs in chondrogenic cells.

## Supporting Information

Figure S1 **Concentration-dependent effects of PACAP on cartilage formation of HDC.** PACAP 1-38 at 10 µM, PACAP 6-38 at 100 nM and 10 µM were administrated continuously from day 1. Metachromatic cartilage areas in 6-day-old cultures were visualized with DMMB dissolved in 3% acetic acid. Metachromatic (purple) structures represent cartilaginous nodules formed by many cells and cartilage matrix rich in polyanionic GAG chains. Original magnification was 4×. Scale bar, 500 µm. Optical density (OD_625_) was determined in samples containing TB extracted with 8% HCl dissolved in absolute ethanol. Representative data of 3 independent experiments are shown.
(PDF)

Figure S2 **Effect of H_2_O_2_ with or without PACAPs on apoptotic rate of cells in 3-day-old HDC.** Cellular viability was determined by FACS analysis. Quadrants 1, 2 and 4 represent cells containing propidium-iodide and/or Annexin V (*i.e.* dead cells), whereas quadrant 3 represents unstained (*i.e.* living) cells of various sizes. Representative data of 3 independent experiments. (C, control; P1-38, PACAP 1-38; P6-38, PACAP 6-38).
(PDF)

Figure S3 **Statistical analysis of RT-PCR and Western blot results of** **Figs. 1** **,** **2** **,** **3** **,** **5** **and** **6** **.** All data are the average of at least three different experiments. Statistical analysis was performed by One Way ANOVA test combined with post hoc tests. All data were normalized on GAPDH and data are expressed as mean ± SEM.
(PDF)
