# Supplementary figures and images for: Pituitary Adenylate Cyclase Activating Polypeptide (PACAP) Signalling Exerts Chondrogenesis Promoting and Protecting Effects: Implication of Calcineurin as a Downstream Target
Source: PLoS One. 2014 Mar 18;9(3):e91541. doi: 10.1371/journal.pone.0091541 (PMC3958376; doi:10.1371/journal.pone.0091541)

## Metachromasia, day 6

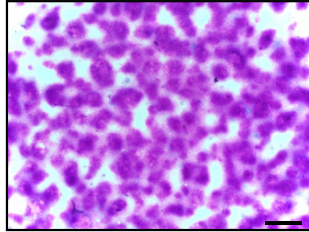

Control  
(OD<sub>625</sub>=100%)

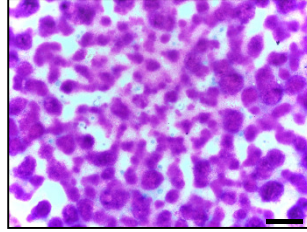

PACAP 1-38 10  $\mu$ M  
(OD<sub>625</sub>=126%\*)

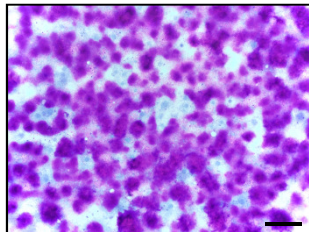

PACAP 6-38 100 nM  
(OD<sub>625</sub>=103%)

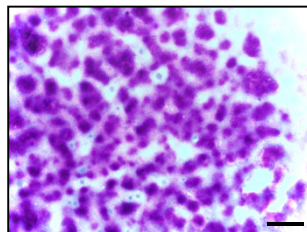

PACAP 6-38 1  $\mu$ M  
(OD<sub>625</sub>=102%)

Supplement: Figure S1 — Concentration-dependent effects of PACAP on cartilage formation of HDC. PACAP 1-38 at 10 µM, PACAP 6-38 at 100 nM and 10 µM were administrated continuously from day 1. Metachromatic cartilage areas in 6-day-old cultures were visualized with DMMB dissolved in 3% acetic acid. Metachromatic (purple) structures represent cartilaginous nodules formed by many cells and cartilage matrix rich in polyanionic GAG chains. Original magnification was 4×. Scale bar, 500 µm. Optical density (OD625) was determined in samples containing TB extracted with 8% HCl dissolved in absolute ethanol. Representative data of 3 independent experiments are shown. (PDF) [file pone.0091541.s001.pdf]

Apoptosis and necrosis, day 3

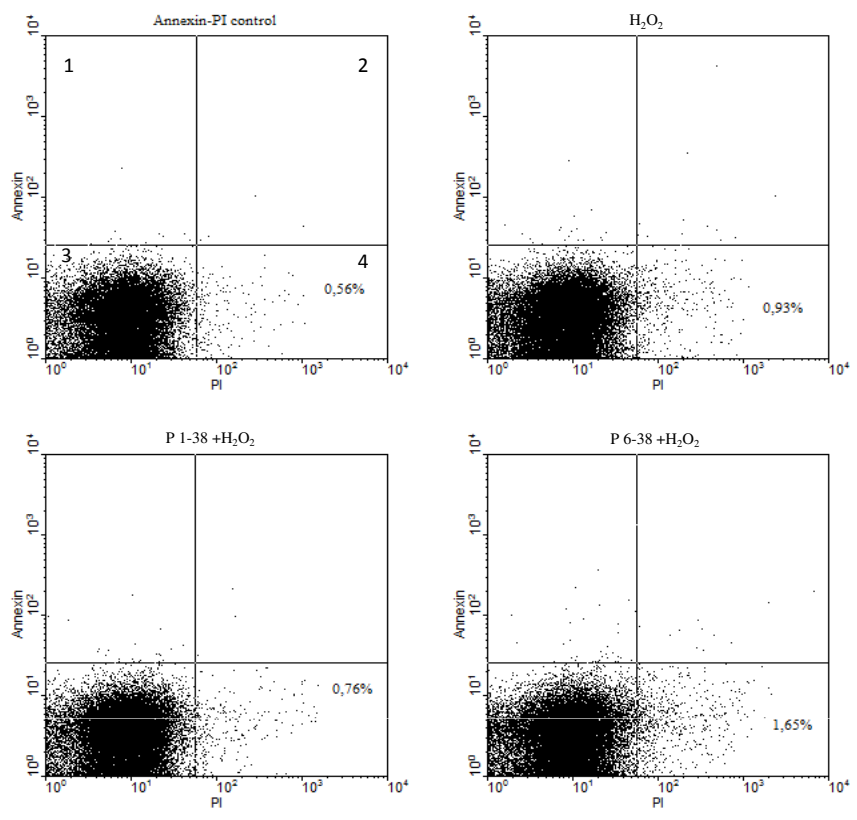

Supplement: Figure S2 — Effect of H2O2 with or without PACAPs on apoptotic rate of cells in 3-day-old HDC. Cellular viability was determined by FACS analysis. Quadrants 1, 2 and 4 represent cells containing propidium-iodide and/or Annexin V (i.e. dead cells), whereas quadrant 3 represents unstained (i.e. living) cells of various sizes. Representative data of 3 independent experiments. (C, control; P1-38, PACAP 1-38; P6-38, PACAP 6-38). (PDF) [file pone.0091541.s002.pdf]
