# Supplementary material for: Pituitary Adenylate Cyclase Activating Polypeptide (PACAP) Signalling Exerts Chondrogenesis Promoting and Protecting Effects: Implication of Calcineurin as a Downstream Target
Source: PLoS One. 2014 Mar 18;9(3):e91541. doi: 10.1371/journal.pone.0091541 (PMC3958376; doi:10.1371/journal.pone.0091541)

# A. Statistical analysis of RT-PCR of Fig 1

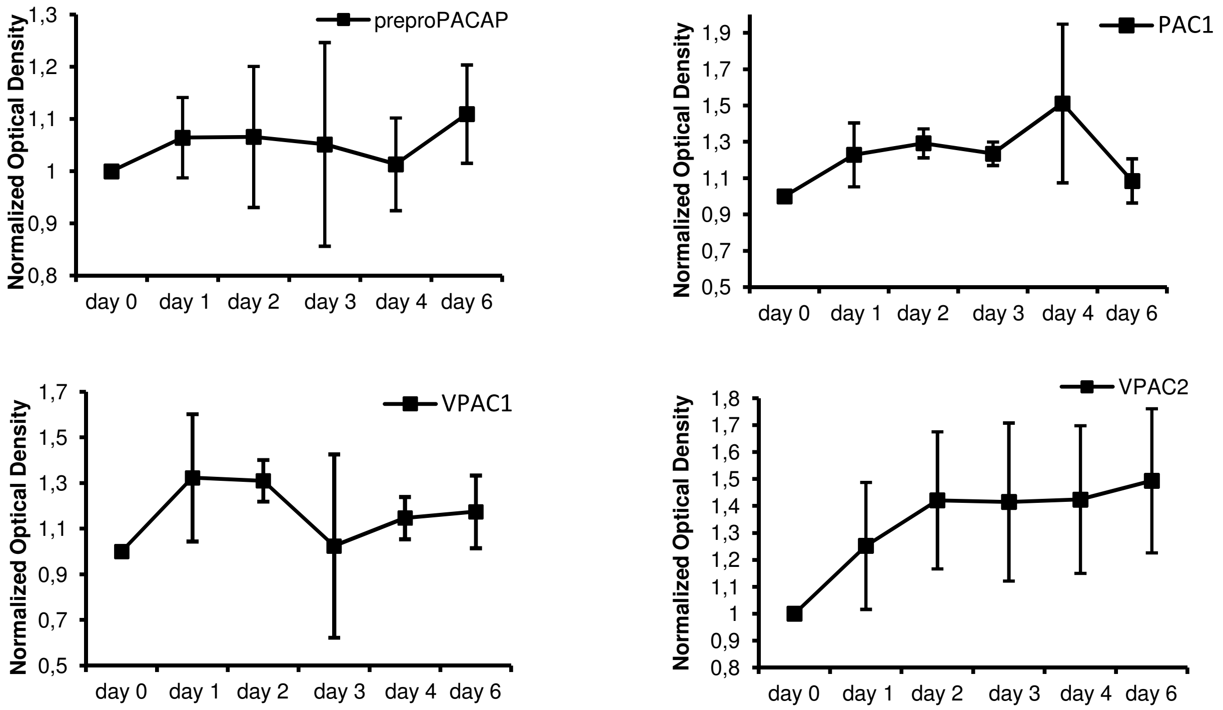


# B. Statistical analysis of Western blot of Fig 1

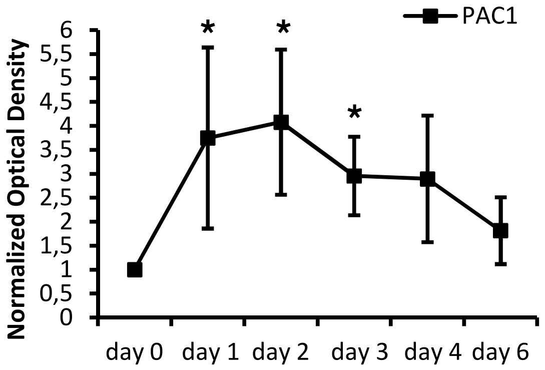

# C. Statistical analysis of RT-PCR of Fig 2

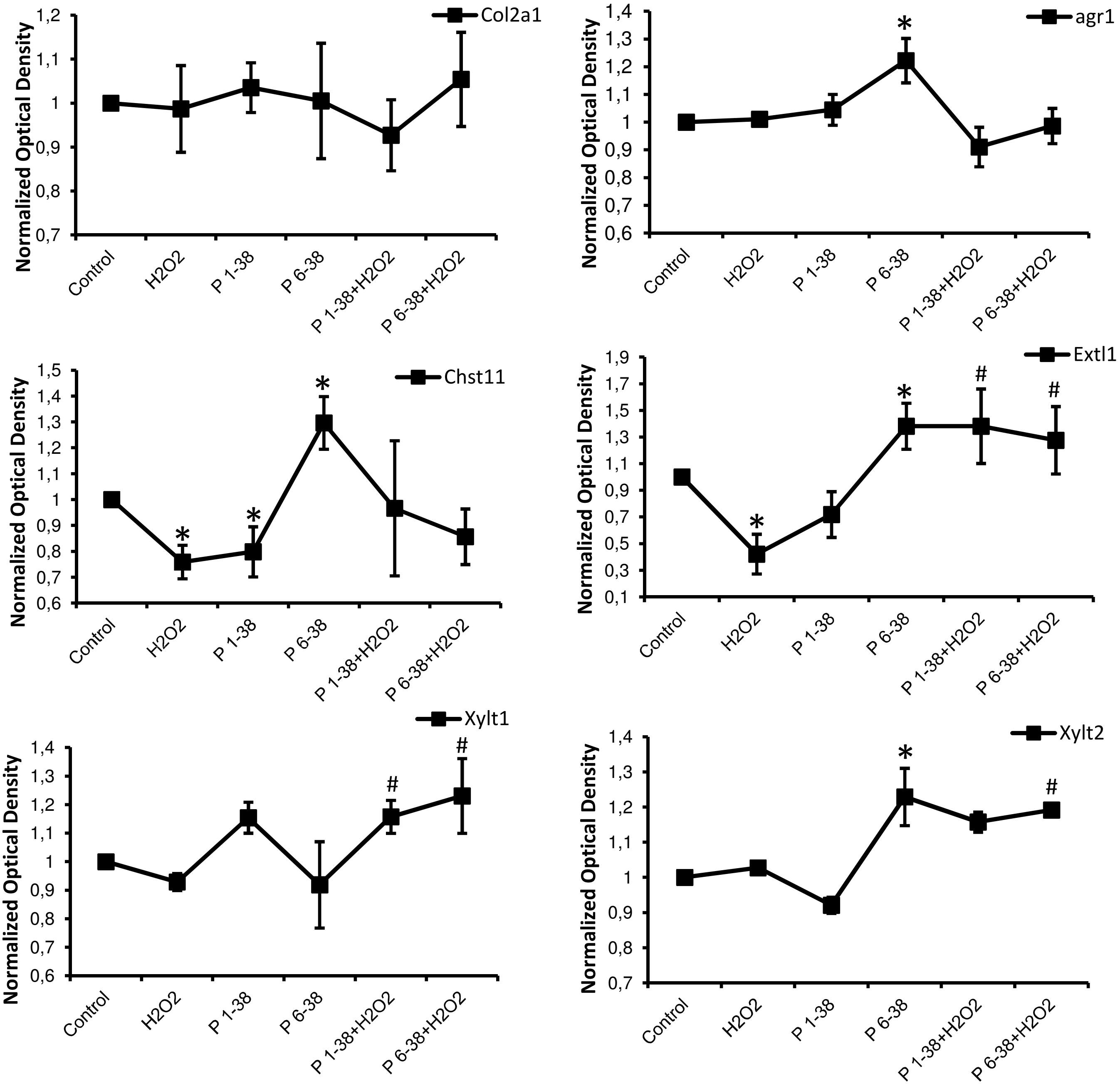

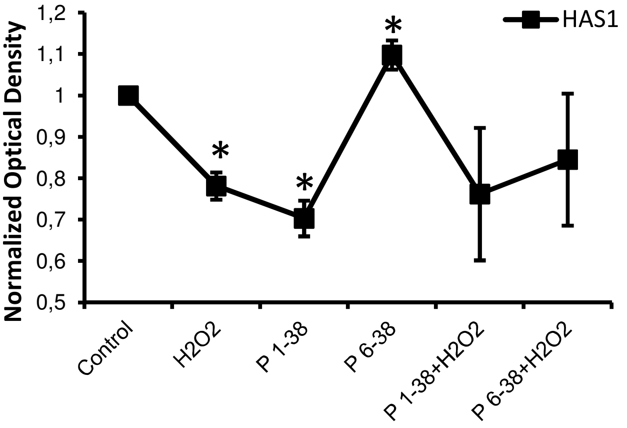

HAS1
Normalized Optical Density
1,2
1,1
1
0,9
0,8
0,7
0,6
0,5
*
*
*
Control
H2O2
P 1-38
P 6-38
P 1-38+H2O2
P 6-38+H2O2


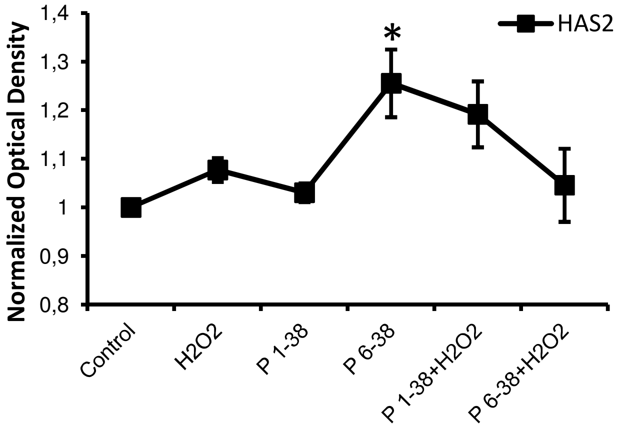

HAS2
Normalized Optical Density
1,4
1,3
1,2
1,1
1
0,9
0,8
*
Control
H2O2
P 1-38
P 6-38
P 1-38+H2O2
P 6-38+H2O2


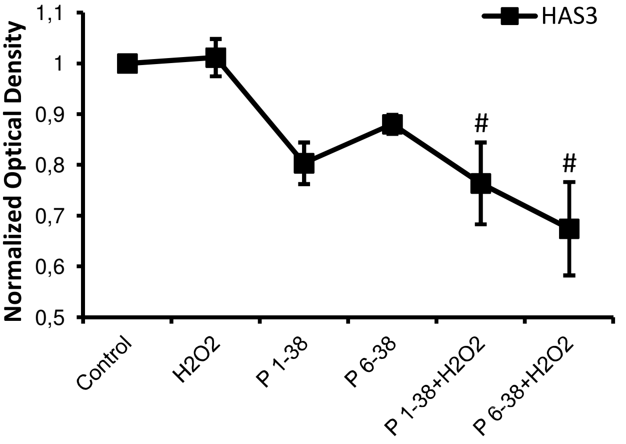

HAS3
Normalized Optical Density
1,1
1
0,9
0,8
0,7
0,6
0,5
#
#
Control
H2O2
P 1-38
P 6-38
P 1-38+H2O2
P 6-38+H2O2

# D. Statistical analysis of Western blot of Fig 2

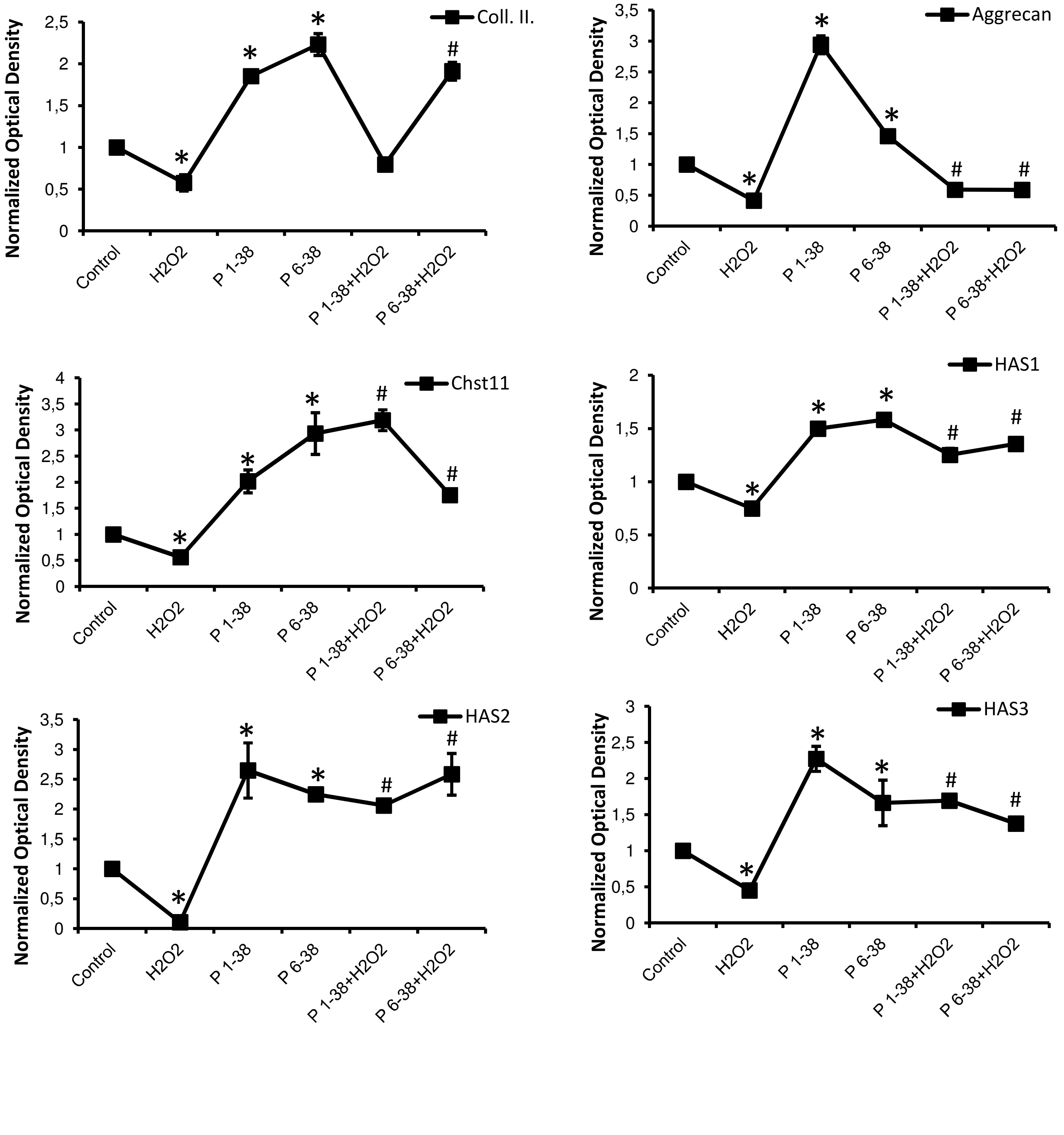

## E. Statistical analysis of RT-PCR of Fig 3

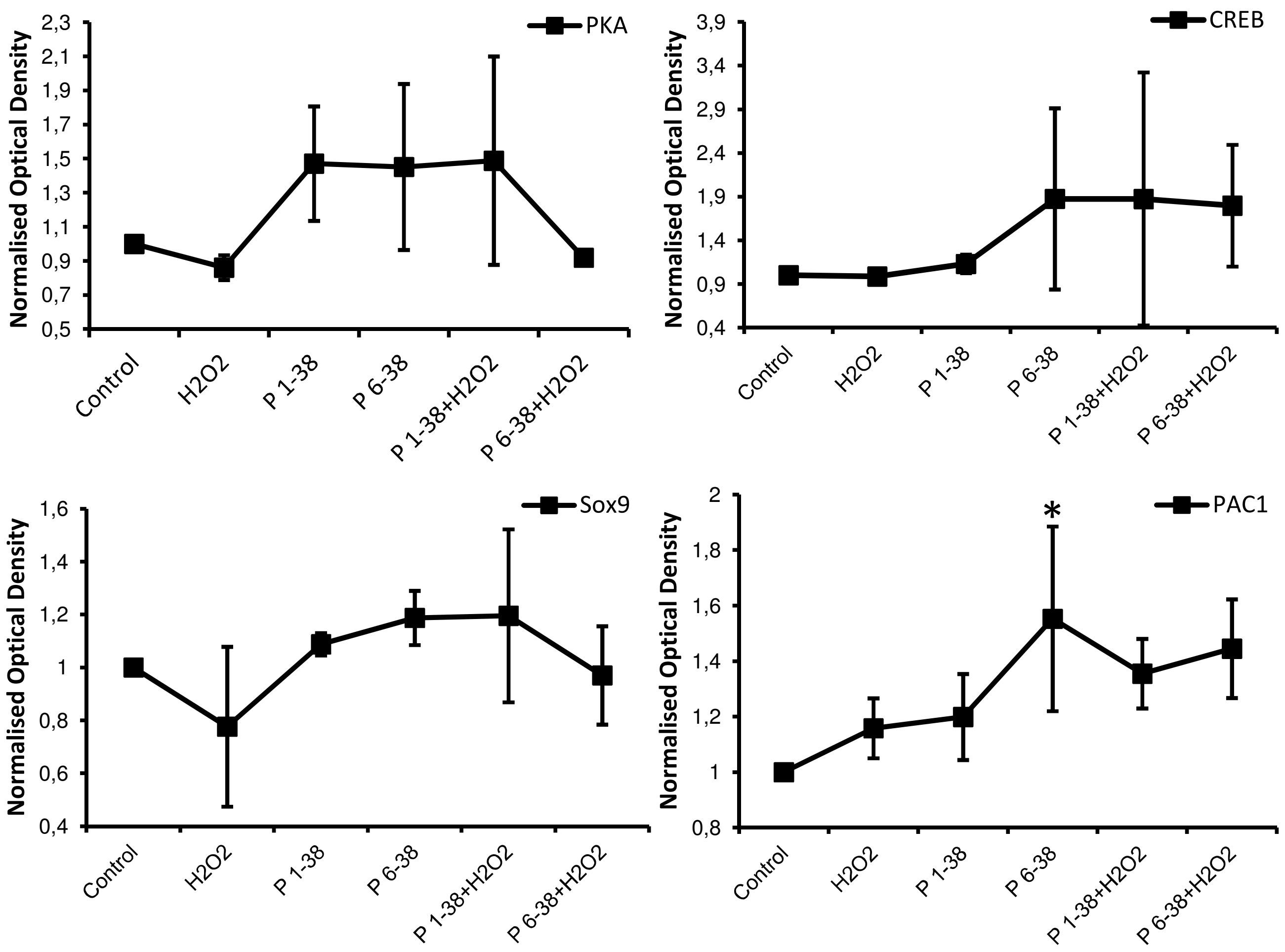

# F. Statistical analysis of Western blot of Fig 3

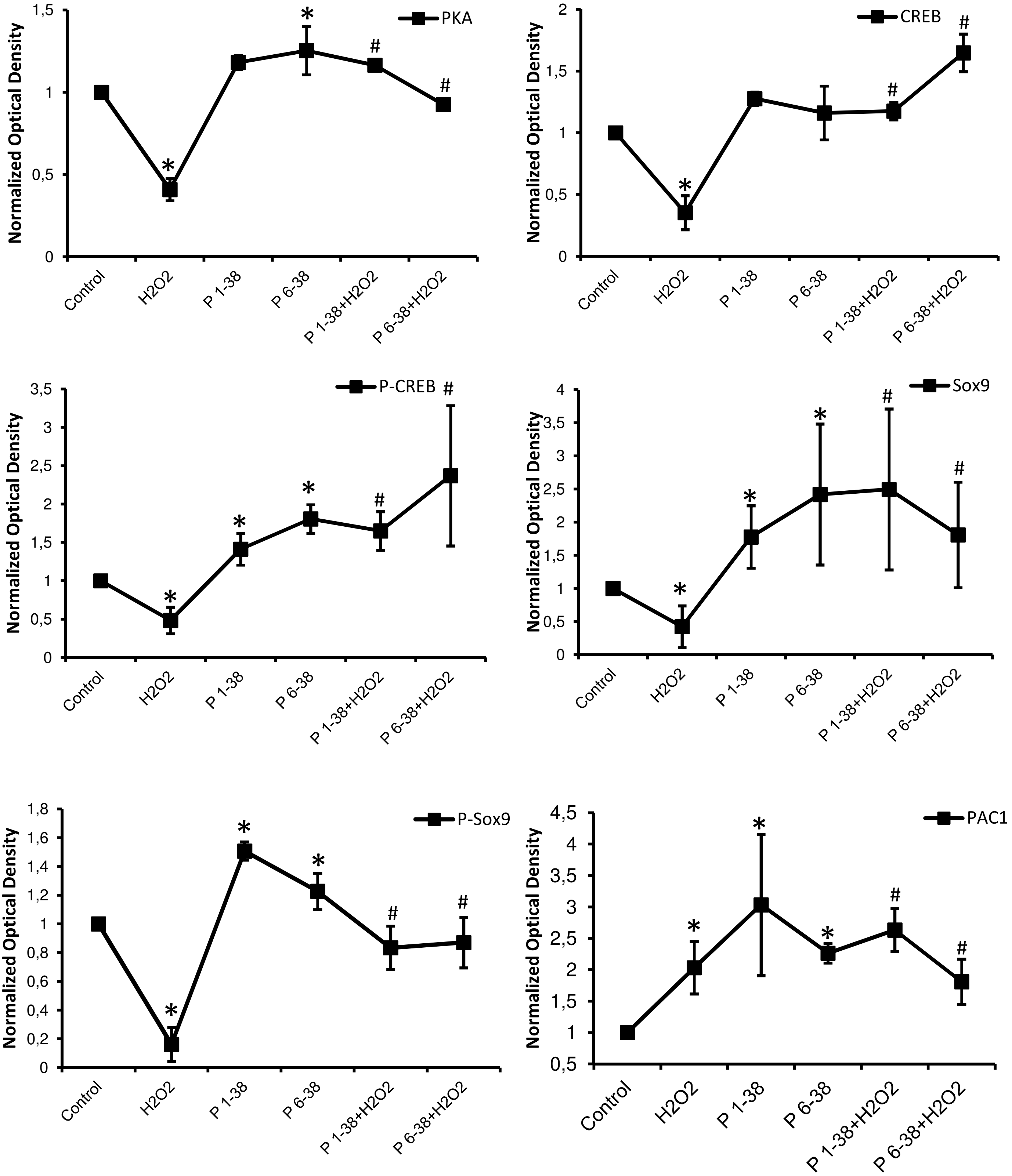

# G. Statistical analysis of RT-PCR of Fig 5

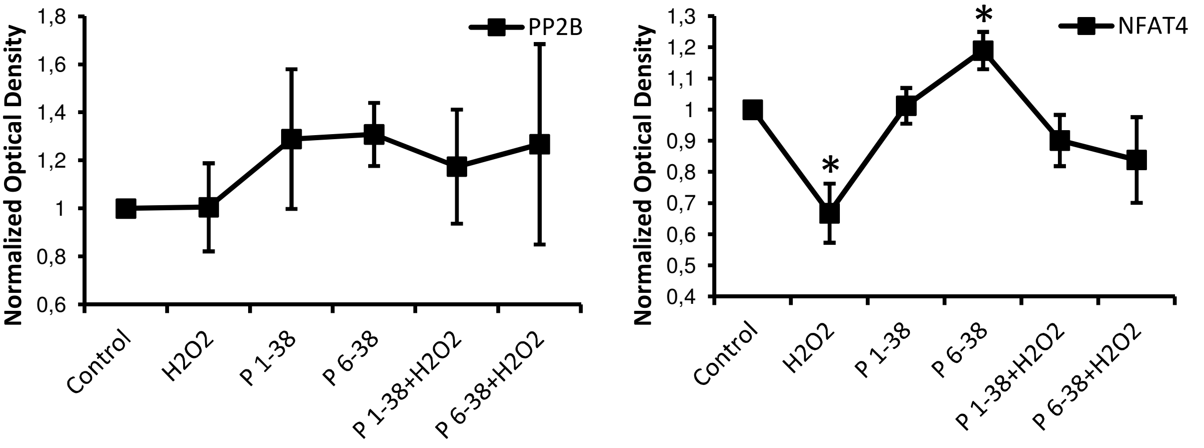


# H. Statistical analysis of Western blot of Fig 5

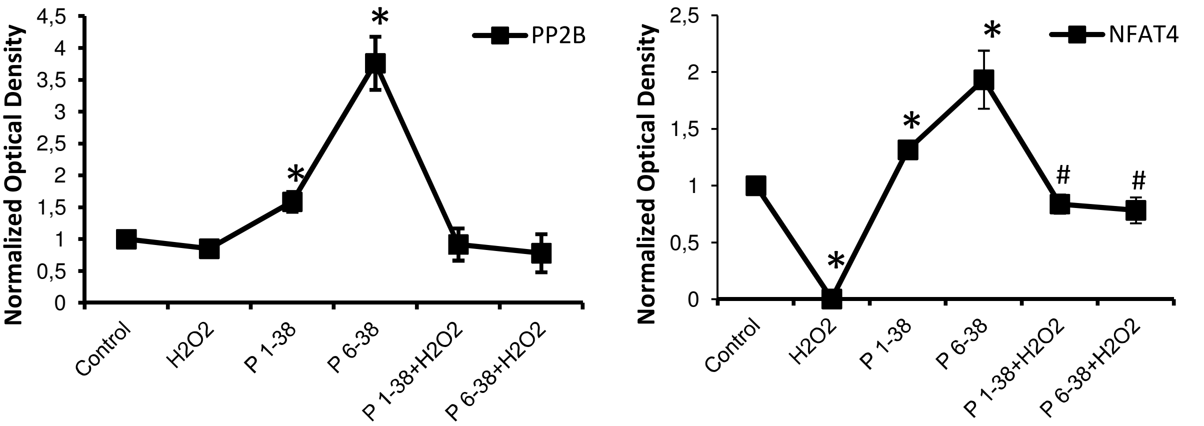

# I. Statistical analysis of RT-PCR of Fig 6

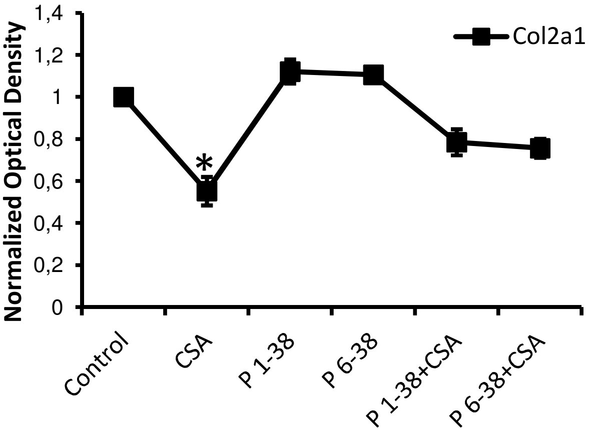


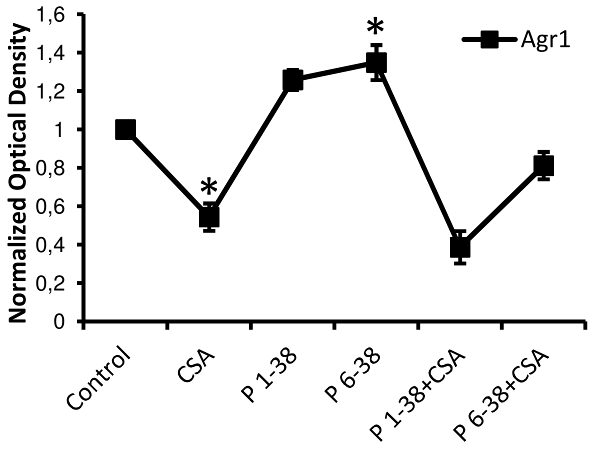


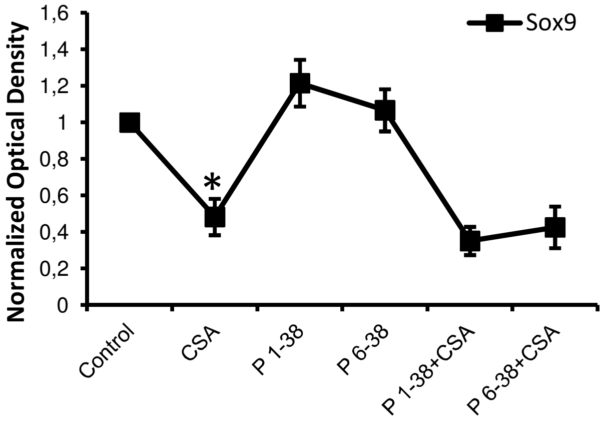

Supplement: Figure S3 — Statistical analysis of RT-PCR and Western blot results of Figs. 1 , 2 , 3 , 5 and 6 . All data are the average of at least three different experiments. Statistical analysis was performed by One Way ANOVA test combined with post hoc tests. All data were normalized on GAPDH and data are expressed as mean ± SEM. (PDF) [file pone.0091541.s003.pdf]
